# Volume staging for arteriovenous malformation SRS treatment using VMAT

**DOI:** 10.1002/acm2.13815

**Published:** 2022-11-10

**Authors:** Claudia Mendez, Ermias Gete

**Affiliations:** ^1^ Department of Physics and Astronomy University of British Columbia Vancouver British Columbia Canada; ^2^ BC Cancer, Abbotsford Center Abbotsford British Columbia Canada; ^3^ BC Cancer, Vancouver Center Vancouver British Columbia Canada

**Keywords:** AVM, Gamma Knife, linac, SRS, volume staging

## Abstract

Volume staging involves dividing the target volume into smaller parts and treating each part separately. In this study, the feasibility of volume‐staged stereotactic radiosurgery (VS‐SRS) on a linear accelerator using volumetric modulated arc therapy (VMAT) and a frameless patient positioning system is investigated. Ten patients, previously treated with hypofractionated stereotactic radiotherapy with arteriovenous malformation (AVM) sized from 1.6 to 4.0 cm in diameter, were selected. VS‐SRS plans were created with the VMAT technique on the Varian Eclipse treatment planning system (TPS) using the TrueBeam STx linear accelerator. For each patient, an AVM‐VMAT set was planned with the AVM as the target and a PTV‐VMAT set using the (PTV = AVM+1 mm) as the target. All targets were divided into two sub‐volumes. The TPS data from the AVM‐VMAT plans was compared to Gamma Knife (GK) VS‐SRS plan data available in the literature. The AVM‐VMAT and PTV‐VMAT plans were compared to investigate the effect of a 1 mm PTV margin on normal brain (NB) dose. End‐to‐end testing was performed using a GaFchromic EBT3 film and point‐dose measurements. Dosimetric effects of multiple setups were investigated through film‐to‐film comparisons. Median target dose coverage, NB *V*_12Gy_, and conformity index for the AVM‐VMAT plans were 97.5%, 17 cm^3^, and 0.8, respectively. PTV‐VMAT plans attained comparable target dose coverage, but the average NB *V*_12Gy_ increased by 48.9% when compared to the AVM‐VMAT plans. Agreement of point‐dose measurements with TPS calculations was −0.6% when averaged over all patients. Gamma analysis passing rates were above 90% for all film‐to‐film comparisons (2%/1 mm criteria), and for the film to TPS comparison (5%/1 mm). This work suggests that VMAT is capable of producing VS‐SRS plans with similar dose falloff characteristics as GK plans. NB dose depends on PTV margin size, and two‐stage treatment setups do not appear to contribute additional uncertainty to treatment delivery.

## 1 INTRODUCTION

Stereotactic radiosurgery (SRS) is an effective treatment option for small (<3 cm) arteriovenous malformations (AVMs) in the brain, and total obliteration of the nidus is achieved with SRS in 70%–95% of patients with small AVMs at 3–10 years.^1^, ^2^, ^3^, ^4^, ^5^ Larger AVMs are more difficult to manage because they are not good candidates for either surgery or radiosurgery. SRS delivered in one fraction to large volumes (>3 cm) has shown lower obliteration rates than those for smaller volumes because lower radiation doses must be used to avoid toxicity to normal tissues, and this compromises the possibility of obliteration.^6^, ^7^, ^8^ In order to be able to deliver an obliteration dose while minimizing the risk of toxicity to normal brain (NB), larger AVMs are treated either with hypofractionated stereotactic radiotherapy (SRT) in which the treatment is delivered in 2–5 fractions^6^ or with volume‐staged SRS (VS‐SRS) in which the AVM is divided into separate stage volumes, and each stage volume is treated with a high dose delivered in a single fraction.^1^, ^3^, ^9^ A time period between the stages is thought to allow for normal tissue repair so that each stage can be treated as a separate target, and higher doses that improve obliteration rate can be delivered.

Several groups using the Gamma Knife (GK) (Elekta AB, Stockholm, Sweden) radiosurgery unit have applied the volume staging approach with satisfactory results.^9^, ^10^, ^11^, ^12^, ^13^, ^14^, ^15^, ^16^, ^17^, ^18^, ^19^, ^20^, ^21^, ^22^, ^23^, ^24^, ^25^, ^26^ A multicenter retrospective review^26^ of patients treated with volume‐staged GK SRS reports cure rates, or no nidus visible in angiography images, of 33.7% and 76.8% for 5 and 10 years of follow‐up. The study included 257 patients with AVM volumes from 7.7 to 94.4 cm^3^ that were treated in 2–4 stages with 12–20 Gy prescribed to the AVM stage volume. This cure rate is comparable to that achieved by single‐stage SRS for lower grade lesions.^20^

Although linear accelerators (LINACs) have proven effective for single and fractionated AVM SRS treatments with similar obliteration rates and toxicity rates as the GK,^28^, ^29^ the application of VS‐SRS to treat large AVMs has primarily been restricted to GK centers. This work aims to evaluate the feasibility of LINAC‐based volume‐staged treatments for large AVMs using volumetric modulated arc therapy (VMAT) and image‐guided patient positioning. The work includes the creation and evaluation of VS‐SRS treatment plans and dosimetric measurements to assess the accuracy of the treatment delivery. This study received ethics approval and was conducted in accordance with institutional regulations.

## 2 MATERIALS AND METHODS

### 2.1 Treatment planning

**FIGURE 1:**
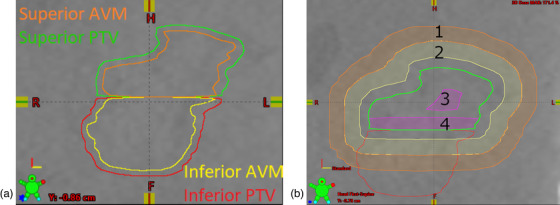
Target and control structures used in arteriovenous malformation (AVM) and PTV volume staging treatment planning in Eclipse. (a) AVM and PTV structures used for patient 8 are shown on a coronal slice of the planning computed tomography (CT) in the specified colors. The AVM volume was divided through the thinner section of the volume to minimize the junction area. (b) Control structures used for the superior stage of patient 8 are shown on the coronal plane of the patient's planning CT. The PTV for the superior stage is shown in green. Structures 1 and 2 are shells created from the PTV at 2 and 5 mm distance from its edge and were used to control the dose falloff from the PTV. Structure 3 was used to keep maximum doses away from the edges of the target, and structure 2 was used to control the dose at the junction of the two stages. Similar structures were also created for the inferior stage.

After a review of the treatment plans of more than 30 AVM patients who were previously treated at our institution with hypofractionated SRT, 10 patients with AVM sizes ranging between 1.6 and 4.0 cm in diameter were chosen for this study. The AVM nidus was delineated on 3D ultrafast gradient echo magnetic resonance and a computed tomography (CT) angiography images and a 1 mm margin was added to create a planning treatment volume (PTV). These planning images and the associated contours of the selected patients were anonymized for planning the volume‐staged treatments. Table 1 shows a summary of the AVM characteristics for the patients included in this study. The staged target volumes were created by dividing the original AVMs into two approximately equal volumes following the strategies adopted by other GK groups.^13^, ^17^, ^18^, ^21^, ^24^ Figure 1a shows the staged AVM and PTV contours for one of the patients in this study. VS‐SRS plans containing 3–5 noncoplanar VMAT arcs were created with the Varian Eclipse (Varian Medical Systems, Palo Alto, California) treatment planning system (TPS) (version 15.6) using the anisotropic analytical algorithm dose calculation algorithm. A Varian TrueBeam equipped with high‐definition multi‐leaf collimator and a six‐degrees‐of‐freedom (6DoF) couch was used for the delivery of the treatment plans.

**TABLE 1:** A summary of the arteriovenous malformation (AVM) characteristics included in this study

Patient	AVM volume (cm^3^)	AVM diameter (cm)	PTV volume (cm^3^)	PTV diameter (cm)	Location	OARs involved
1	34.6	4.0	45.8	4.4	Parietal	None
2	30.9	3.9	44.6	4.4	Temporal	Brainstem, optics
3	7.8	2.5	11.0	2.8	Parietal	None
4	3.8	1.9	6.1	2.3	Temporal	None
5	3.1	1.8	5.2	2.2	Temporal	Brainstem
6	20.3	3.4	27.4	3.7	Parietal	None
7	14.8	3.0	22.0	3.5	Occipital	None
8	6.8	2.3	9.8	2.7	Temporal	Brainstem
9	2.2	1.6	3.7	1.9	Temporal	Brainstem, optics
10	25.3	3.6	34.2	4.0	Temporal	Brainstem

Abbreviation: OARs, organs at risk.

For each patient, two sets of VS‐SRS plans were created. The difference between the two sets of plans was the margin used to create the staged planning target volume. The first set, called AVM‐VMAT, was planned with the AVM itself set to be the PTV, following the GK planning method. For the second set, a PTV was created by allowing a 1 mm margin over the AVM in accordance with the current practice of LINAC‐based SRS at our center. These plans are called PTV‐VMAT plans. The AVM‐VMAT plans were created to investigate whether VMAT is capable of producing dosimetrically comparable plans to GK plans. The AVM‐VMAT plans also served as a reference for evaluating the increase in normal tissue doses due to the addition of the 1 mm margin.

A single fraction dose of 20 Gy was prescribed to each staged volume. Dose constraints for organs at risk (OARs) were set following recommendations of the AAPM TG‐101.^29^ In addition, the recommendation of the HyTEC project^30^ for AVM SRS to limit the volume of NB tissue that receives 12 Gy or more, or NB *V*_12Gy_, to 10 cm^3^ was used. A summary of the target and OAR dose constraints that were used for planning each stage is included in Table 2.

**TABLE 2:** Summary of the dose constraints used for the 20 Gy/1 fraction prescription used for treatment planning

Structure	Dosimetric parameter	Constraint
Optics	*D*_max_ $D_{0.2cm^{3}}$	≤10 Gy ≤8 Gy
Cord	*D*_max_ $D_{0.5cm^{3}}$	≤14 Gy ≤10 Gy
Brainstem	*D*_max_ $D_{0.35cm^{3}}$	≤15 Gy ≤10 Gy
Target	*V*_95%_ *D*_max_	≥99% ≤150%
Retina	*D*_max_	≤2 Gy
Anterior chamber	*D*_max_	≤1 Gy
Normal brain	*D*_max_ *V*_12Gy_	≤21 Gy ≤10 cm^3^

*Note*: These are nominal values that should not be surpassed, which is possible when a structure is abutting the target volume.

Figure 1b shows the control regions created to minimize the dose at the junction and to encourage a sharp drop of dose outside the target. Each stage had two 3 mm thick control shells at a distance of 2 and 5 mm from the target with max dose constraints set at around 80% and 50%, respectively. A control structure made of a 2–4 mm thick section of the PTV stage at the junction was used to limit the maximum dose in this region to the prescription dose. Additionally, an optimization structure was created by creating a negative margin of 3–5 mm inside the target. This structure was used to force the location of maximum dose to the center of the treated volume. Finally, the NB, which is the brain minus the staged AVM volume, was created to further control the dose falloff outside the target. A minimum of three noncoplanar VMAT arcs were used, and an effort was made to avoid nearby critical structures and the untreated AVM volume when selecting the arc angle.

Each volume‐staged plan created in this study was evaluated individually for maximum dose (*D*_max_) inside the target, conformity, prescription coverage, NB *V*_12Gy_, *D*_max_ to NB, and OAR doses. The composite plans were evaluated in terms of PTV *D*_max_ and *V*_40Gy_, as recommended by McKay et al.,^31^ for cumulative doses of repeated radiotherapy to the brain. Conformity of the prescription dose to the target volume was evaluated using the Paddick conformity index (CI).^32^ The staged AVM‐VMAT plans were compared with the PTV‐VMAT plans and the dosimetric consequences of using a 1 mm margin on the PTV was investigated.

### 2.2 End‐to‐end testing

**FIGURE 2:**
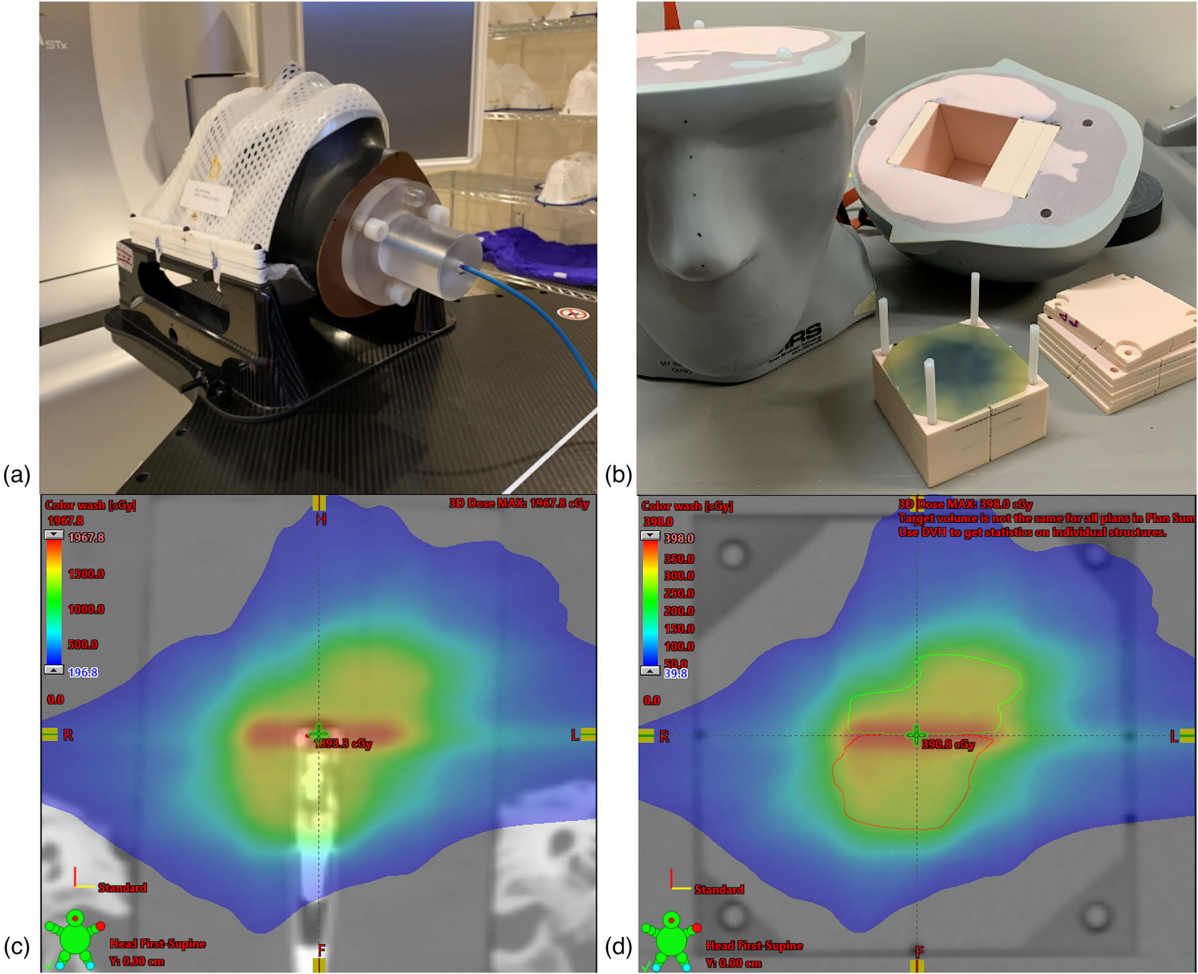
Phantoms used for dosimetric verification. (a) Head phantom with custom acrylic insert for the microDiamond detector. (b) CIRS head phantom with film insert showing the central plane where the film was placed. The grooves on the slabs are used for marking the fiducials on the film. (c) The dose distribution of patient 8 of the composite plan calculated along the coronal plane on the microDiamond phantom. Note the detector's sensitive volume is at the junction between the two stages. (d) Dose distribution of the same plan calculated on the CIRS phantom for film dosimetry.

End‐to end testing was performed to measure the accuracy of the individual and the composite dose delivery of each volume‐staged plan. The tests were performed using two anthropomorphic head phantoms, as shown in Figure 2. The phantom shown on the left in Figure 2a was made by Phantom Laboratories, USA and had an acrylic insert for point‐dose measurement with the microDiamond detector (PTW, model 60019). The detector was placed at the center of the overall AVM volume, so the dose was measured at the junction of the two stages, as shown in Figure 2c.

The phantom on the right, Figure 2b, is a CIRS. Inc. (Norfolk, VA) Radiosurgery Test Phantom, model 605, with a film insert, and was used for 2D film measurements of the composite dose delivery. A radiochromic film, type GaFchromic EBT3 (Ashland Inc., Covington, KY), was used to measure dose distributions in the phantom in a plane that included the junction area, as shown in Figure 2d. The radiochromic film was calibrated following the recommended procedure for radiotherapy use by the AAPM TG‐235 report.^33^ The film handling and protocol proposed by Lewis et al.^34^ was followed. For each measurement, one film was placed in the midplane of the film holder and aligned with the coronal plane of the CIRS phantom.

**FIGURE 3:**
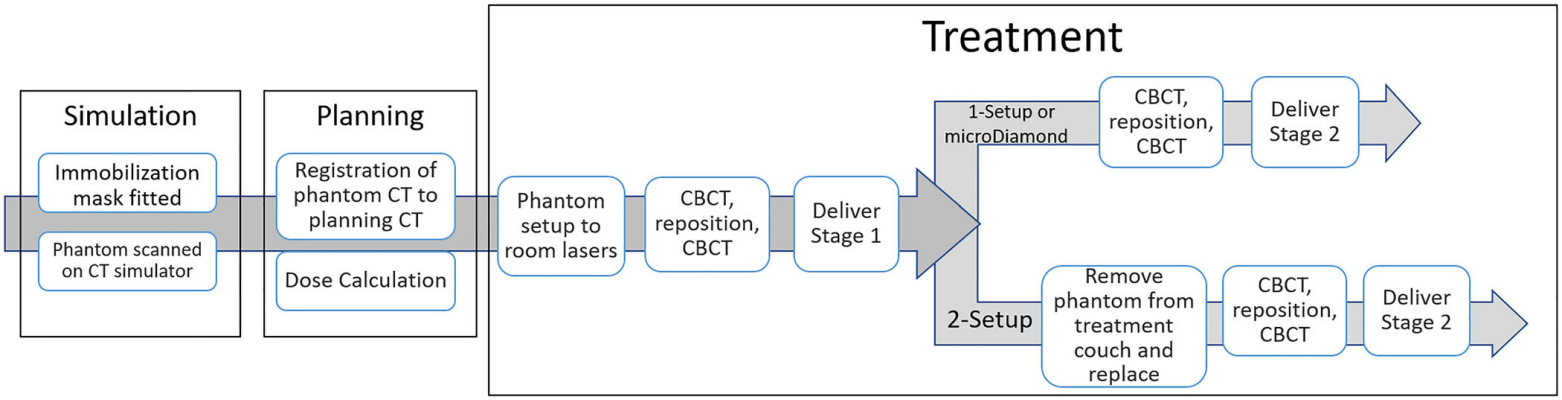
Flowchart of the end‐to‐end testing process showing how the point dose, the 1‐setup and the 2‐setup film measurements were acquired

Figure 3 depicts the workflow used for the end‐to‐end testing. Both phantoms were fitted with a frameless stereotactic mask for the Brainlab frameless SRS universal couch extension (Brainlab AG, Munich, Germany) and were scanned on a CT simulator using the scan protocol for SRS treatments at our center, 0.63 mm slice thickness, and 0.74 mm pixel size. Each volume‐staged plan was exported to both phantoms and calculated for delivery on the LINAC. SRS patient treatment setup procedures were followed during phantom setup for treatment delivery. The onboard cone beam CT and the Varian 6DoF couch were used for positioning the phantoms.

#### 2.2.1 Composite dose delivery verification with a film

Two separate film measurements were performed for each pair of staged treatment plans. Figure 3 details the treatment workflow that was followed for these measurements. For the first group of measurements, a single film was exposed to the treatment of both stages, whereas the phantom was positioned on the couch only once at the start of the measurement. The films from this setup are termed “1‐setup” films. For the second group, the phantom was removed from the couch extension and repositioned before the second stage delivery to simulate the setup of an actual staged treatment where the patient would return at a later date for a second treatment. The films obtained from this measurement are termed “2‐setup” films.

Each film was scanned on an Expression 10000 XL (Epson, Long Beach, CA) scanner using 72 dpi, 48‐bit color, and transmission mode. The scanned film data was imported to the Film QA Pro 2014 (Ashland, Inc., Covington, KY) software for analysis. The measured dose distributions from the 1‐setup and 2‐setup films were compared to each other to investigate whether an additional error is introduced by repositioning the phantom for the second delivery. Additionally, each of the film measurements was compared to the absolute dose calculated by the TPS. The fiducials cut in the film were used to register the films to each other and to localize the isocenter.

The dose distributions were evaluated using gamma analysis, dose profiles, isodose lines and dose difference maps. The chosen pass criteria for the gamma analyses of the film‐to‐film comparisons were 3%/1 mm, 2%/1 mm, and 1%/1 mm for all pixels. Gamma analysis pass criteria of 5%/1 mm and 3%/1 mm were used for the comparisons of film measurements to the TPS.

#### 2.2.2 Point‐dose measurement

Point‐dose measurements were performed with a microDiamond detector placed at the junction between the two stages (Figure 2c). The workflow that was followed is shown in Figure 3, and it is similar to the 1‐setup film measurement. The charge collected by the microDiamond for each treatment arc was recorded, and the reading was converted to dose using a dose calibration factor that was based on an ion chamber measurement of a 4 cm by 4 cm reference field. Finally, the percent difference between the calculated point dose and the measured dose was calculated.

## 3 RESULTS

### 3.1 Treatment planning

#### 3.1.1 AVM‐VMAT plans

**FIGURE 4:**
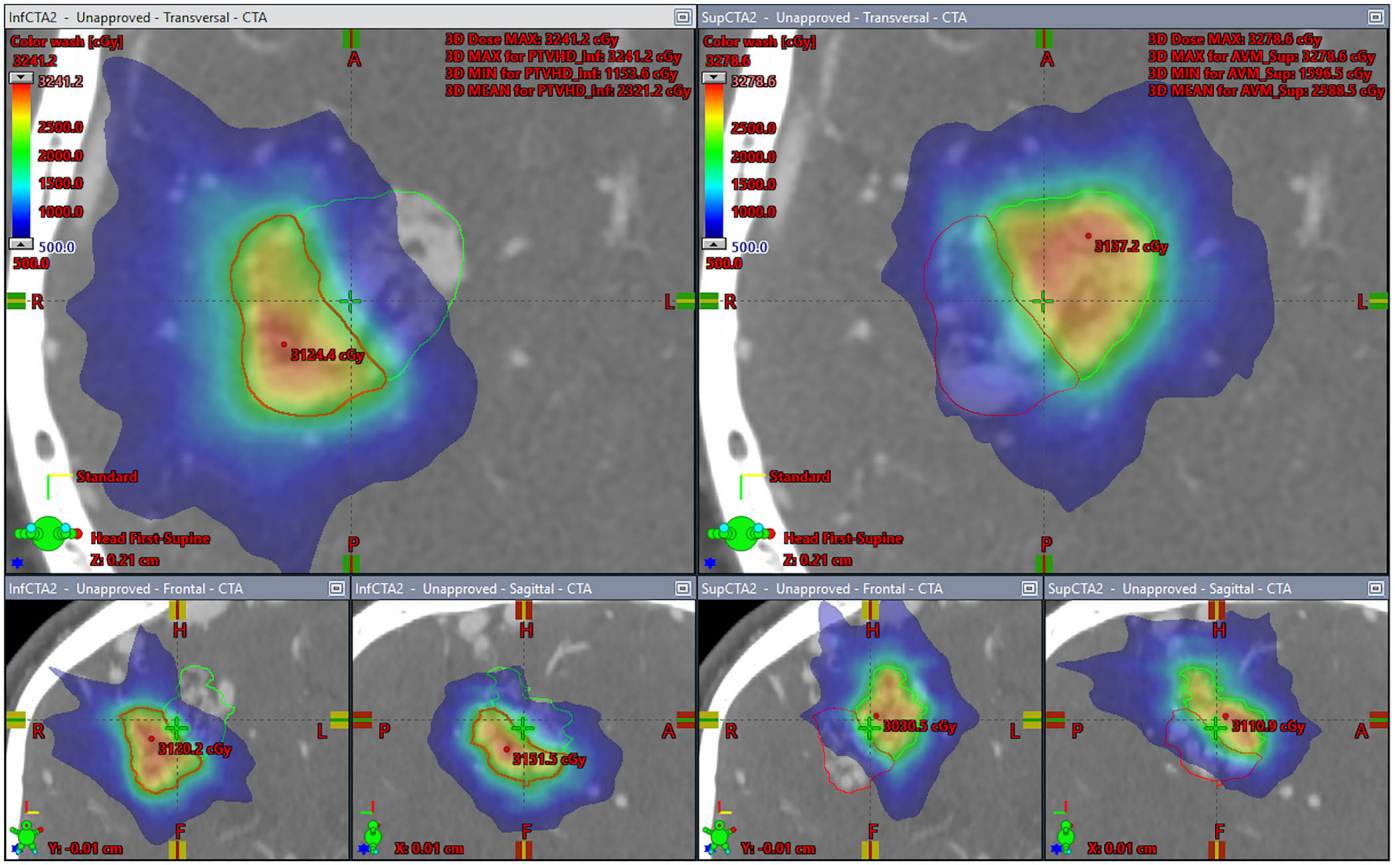
Dose distributions for patient 3 from the AVM‐VMAT volume‐staged plans calculated with Eclipse for the inferior stage (left) and superior stage (right). The superior AVM is shown in green and the inferior AVM in red.

**FIGURE 5:**
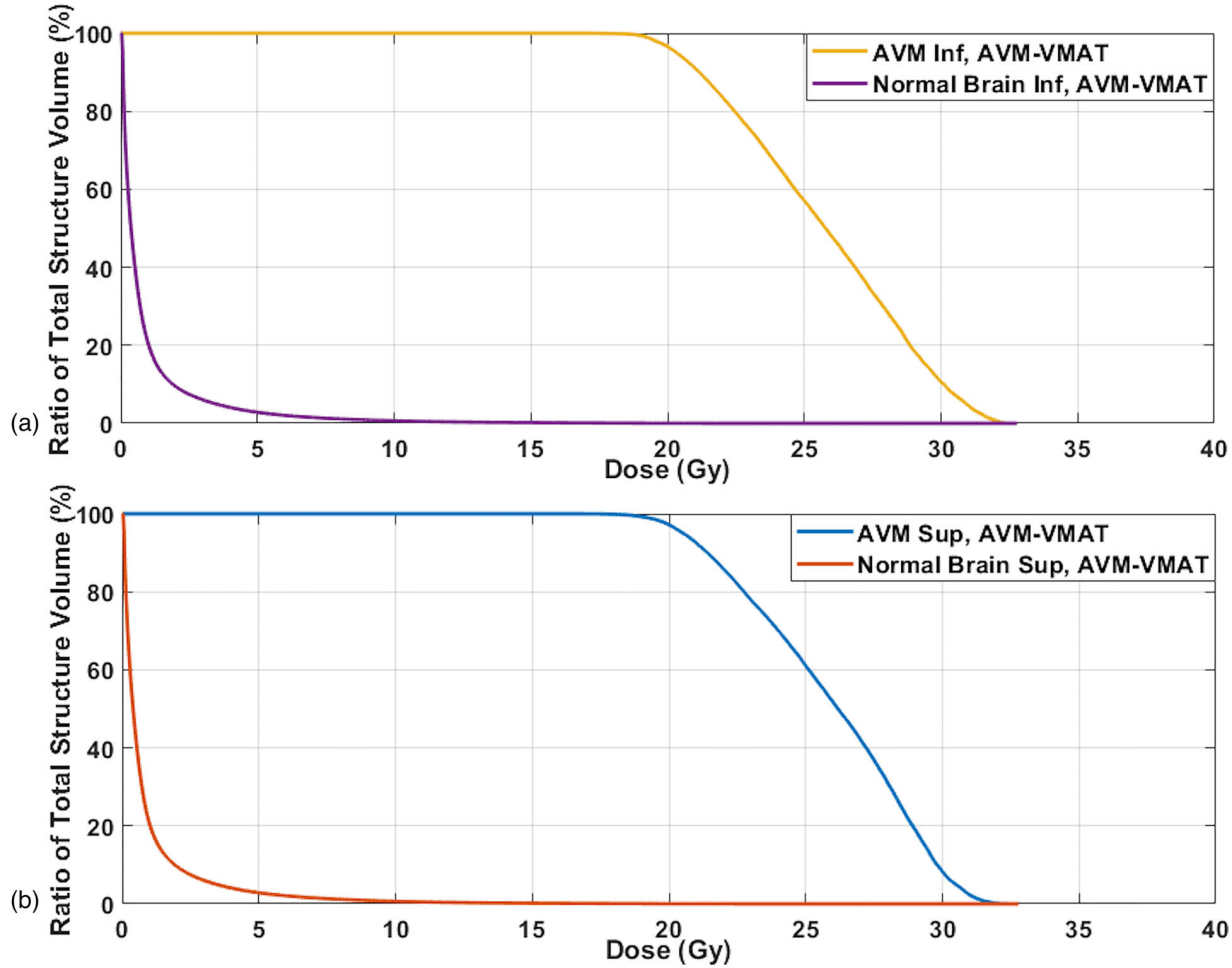
Graphs showing the arteriovenous malformation (AVM) and normal brain (NB) DVHs from AVM‐VMAT staged plans for patient 3: (a) inferior stage and (b) superior stage

**FIGURE 6:**
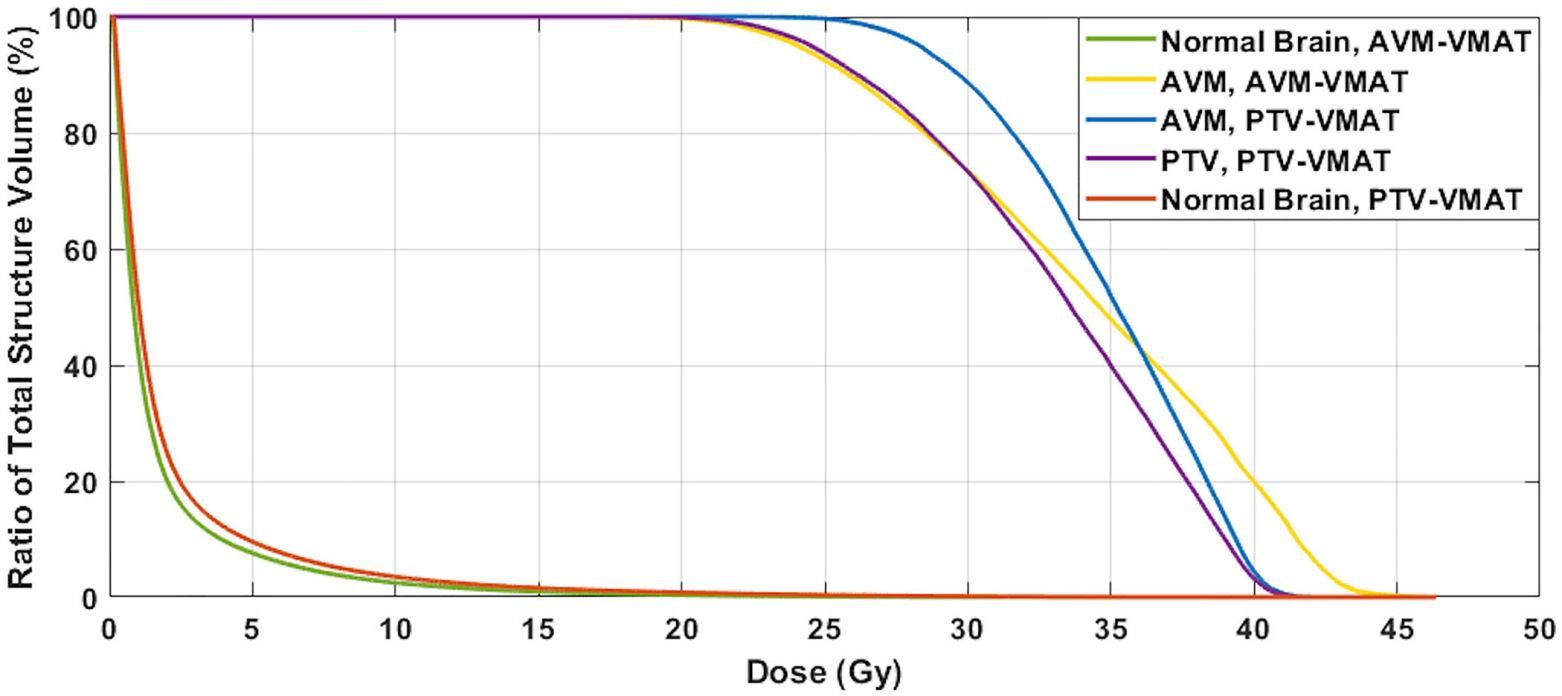
Graphs comparing the composite normal brain and target doses for the arteriovenous malformation AVM‐VMAT and PTV‐VMAT plans for patient 3. The DVHs for the PTV are shown only for the PTV‐VMAT plan. The PTV‐VMAT DVHs are shown in red for the normal brain, purple for the PTV, and blue for the AVM.

Figure 4 shows the dose distributions for the staged AVM‐VMAT plans of patient 3. The figure on the right shows the distributions from the plan that treats the superior portion of the AVM highlighted with the green contour, and the figure on the left shows the distributions treating the inferior portion of the AVM represented by the red contour. As can be seen from the figure, the region of high dose was confined to the treated AVM and away from the junction of the two stages. The NB DVHs of the same staged plans are shown in Figure 5. The maximum dose to the treated AVM was 164% and 162% for the superior and inferior stages with the prescription dose covering more than 96% of the each stage's volume. The DVHs for the composite plan are shown in Figure 6. The green and the yellow curves represent the DVHs for the NB and the AVM, respectively.

Dosimetric data from the AVM‐VMAT plans for all the 10 patients is listed in Table 3. The target coverage evaluation, including the maximum dose (*D*_max_) inside the target, the target volume covered by the prescription isodose, and the CI are listed in columns 4–6 of Table 3. Dose to OARs, which includes *D*_max_ to NB, *V*_12Gy_ for NB, and doses to the brainstem and the optics are also given.

**TABLE 3:** Dosimetric results of the AVM‐VMAT volume‐staged treatment plans

Patient	Stage	Volume (cm^3^)	Stage *D*_max_ (Gy)	Stage *V*_20Gy_ (%)	CI	Normal brain *D*_max_ (Gy)	Normal brain *V*_12Gy_ (cm^3^)	Brainstem $D_{0.35cm^{3}}$ (Gy)	Optics $D_{0.22cm^{3}}$ (Gy)
1	Sup	17.3	32.9	95.6	0.87	26.6	20.6	1.2	1.6
	Inf	17.0	32.6	94.1	0.88	25.7	21.2	1.0	0.9
2	Sup	19.0	28.4	95.5	0.86	24.8	34.4	4.0	4.2
	Inf	11.6	27.2	83.5	0.65	23.5	21.6	16.0	16.0
3	Sup	3.4	32.8	97.2	0.86	27.5	4.9	1.5	0.5
	Inf	3.9	32.4	96.6	0.91	24.3	5.1	0.8	0.3
4	Lat	2.1	30.0	97.6	0.80	28.0	4.9	1.9	0.8
	Med	1.6	30.6	98.8	0.76	25.3	4.2	2.5	1.0
5	Ant	1.6	25.6	97.3	0.74	24.2	5.5	7.5	1.4
	Post	1.4	24.6	98.8	0.64	21.9	4.5	4.6	7.8
6	Sup	10.3	27.4	99.6	0.89	25.9	14.6	0.6	0.5
	Inf	9.7	26.7	92.7	0.79	25.7	15.4	2.4	2.8
7	Sup	7.0	27.3	99.5	0.76	23.3	9.8	2.8	2.0
	Inf	7.5	26.8	98.2	0.88	25.0	13.2	2.9	1.2
8	Sup	3.4	27.6	99.3	0.69	24.7	10.6	1.7	1.3
	Inf	3.3	29.7	99.9	0.78	25.2	8.2	5.3	2.0
9	Sup	1.0	27.8	97.0	0.73	25.2	2.9	2.4	1.5
	Inf	1.1	26.1	99.2	0.81	25.8	3.0	5.6	3.3
10	Sup	12.4	27.6	98.7	0.91	24.8	18.4	5.5	3.5
	Inf	12.6	28.2	91.7	0.85	26.0	18.1	6.0	2.2

Abbreviation: CI, conformity index.

The overall results for the AVM‐VMAT plans for all 10 patients show that all stages had over 91% coverage of the AVM by the prescription dose, except for the inferior stage of patient 2, which had a coverage of 83.5% due to its overlap with the brainstem. When averaging over all the stages, the AVM maximum dose was 28.6 Gy, the prescription coverage of the AVM was 96.5%, and the CI was 0.80.

The maximum dose to the NB and the NB *V*_12Gy_ had average values of 25.2 Gy and 12.0 cm^3^, respectively. Five of the 10 plans surpassed the *V*_12Gy_ constraint of 10 cm^3^. OAR constraints were not violated, with the exception of the inferior stage for patient 2 due to its proximity to the brainstem and the optics. The *V*_40Gy_ for the composite plans was below the constraint of 0.28 cm^3^ for all patients, except for patient 3.

#### 3.1.2 PTV‐VMAT plans

The PTV‐VMAT plans had the same characteristics as the AVM‐VMAT plans except for the increased NB *V*_12Gy_ volume. Figure 6 shows the DVH graphs comparing the AVM‐VMAT and PTV‐VMAT plans for the composite plans for patient 3 as an example. It should be noted that NB here is defined as before: the brain minus the staged AVM volume. The increase in the NB dose is apparent as well as the higher coverage of the AVM volume by the PTV‐VMAT plans. Table 4 shows the results of the analysis of the 10 PTV‐VMAT volume staging plans prepared for this study. For target coverage evaluation, the target volume covered by the prescription isodose, the maximum dose (*D*_max_) inside the target and the CI are listed. Dose to OARs, which includes *D*_max_ in NB outside the AVM, *V*_12Gy_ for NB, and doses to any critical structures near the target are also shown.

**TABLE 4:** Dosimetric results of the PTV‐VMAT volume‐staged treatment plans

Patient	Stage	Volume (cm^3^)	Stage *D*_max_ (Gy)	PTV stage *V*_20Gy_(%)	AVM stage *V*_20Gy_ (%)	CI	Normal brain *D*_max_ (Gy)	Normal brain *V*_12Gy_ (cm^3^)	Brainstem $D_{0.35cm^{3}}$ (Gy)	Optics $D_{0.22cm^{3}}$ (Gy)
1	Sup	22.1	35.5	91.9	97.0	0.89	27.5	24.2	0.9	1.1
	Inf	21.5	32.9	91.0	97.8	0.90	27.9	27.8	1.3	1.0
2	Sup	25.7	29.6	94.6	98.9	0.93	28.1	43.5	3.8	4.2
	Inf	18.6	23.1	67.7	82.4	0.64	22.7	37.5	16.5	12.4
3	Sup	5.4	35.9	92.7	97.8	0.87	30.7	9.4	1.9	0.7
	Inf	5.6	32.8	95.9	99.2	0.90	28.8	9.2	0.9	0.3
4	Lat	3.3	34.3	95.0	96.5	0.87	30.8	7.0	2.1	1.1
	Med	2.6	34.7	92.7	98.7	0.88	28.3	5.7	2.8	1.3
5	Ant	2.7	25.5	98.9	99.8	0.84	24.7	8.0	8.4	1.7
	Post	2.5	28.2	94.1	93.6	0.78	27.6	7.2	6.0	0.8
6	Sup	13.7	28.6	96.2	97.9	0.95	27.3	18.1	1.1	0.8
	Inf	13.6	28.8	92.4	93.3	0.82	28.0	21.7	1.9	2.7
7	Sup	11.0	27.9	97.2	99.5	0.92	26.9	17.6	2.4	1.8
	Inf	10.8	27.2	93.6	97.1	1.05	26.1	15.1	2.7	1.2
8	Sup	5.0	34.3	97.0	97.3	0.82	28.8	12.6	1.5	0.6
	Inf	4.7	32.1	98.1	99.1	0.89	27.4	12.1	6.9	2.5
9	Sup	1.7	28.5	90.6	95.7	0.82	27.0	3.9	2.5	1.9
	Inf	1.9	29.4	96.9	100.0	0.91	28.6	4.5	6.9	4.7
10	Sup	16.8	28.2	99.6	99.9	0.88	25.6	36.6	7.7	5.2
	Inf	17.1	30.0	95.6	97.0	0.89	26.2	33.2	7.3	2.5

Abbreviations: AVM, arteriovenous malformation; CI, conformity index.

All plans had over 90% coverage of the PTV and over 93% coverage of the AVM by the prescription dose, except for the inferior stage of patient 2 due to its overlap with the brainstem. PTV coverage was dependent on the location of the AVM, and the coverage in some cases was compromised due to its proximity to critical structures. When averaging over all the stages, the PTV maximum dose was 30.4 Gy, the prescription coverage of the PTV and AVM were 93.6% and 96.9%, respectively. The average CI was 0.87 and the average NB maximum dose was 27.4 Gy.

As expected, the magnitude of *V*_12Gy_ was highly correlated with the volume of the target, and its constraint could not be met for 7 of the 10 patients. The brainstem and optics dose constraints were not met only for one case, the inferior stage for patient 2, due to the proximity of the PTV to both structures. Unlike the AVM‐VMAT plans, the *V*_40Gy_ constraint was surpassed by 4 of the 10 patients.

**FIGURE 7:**
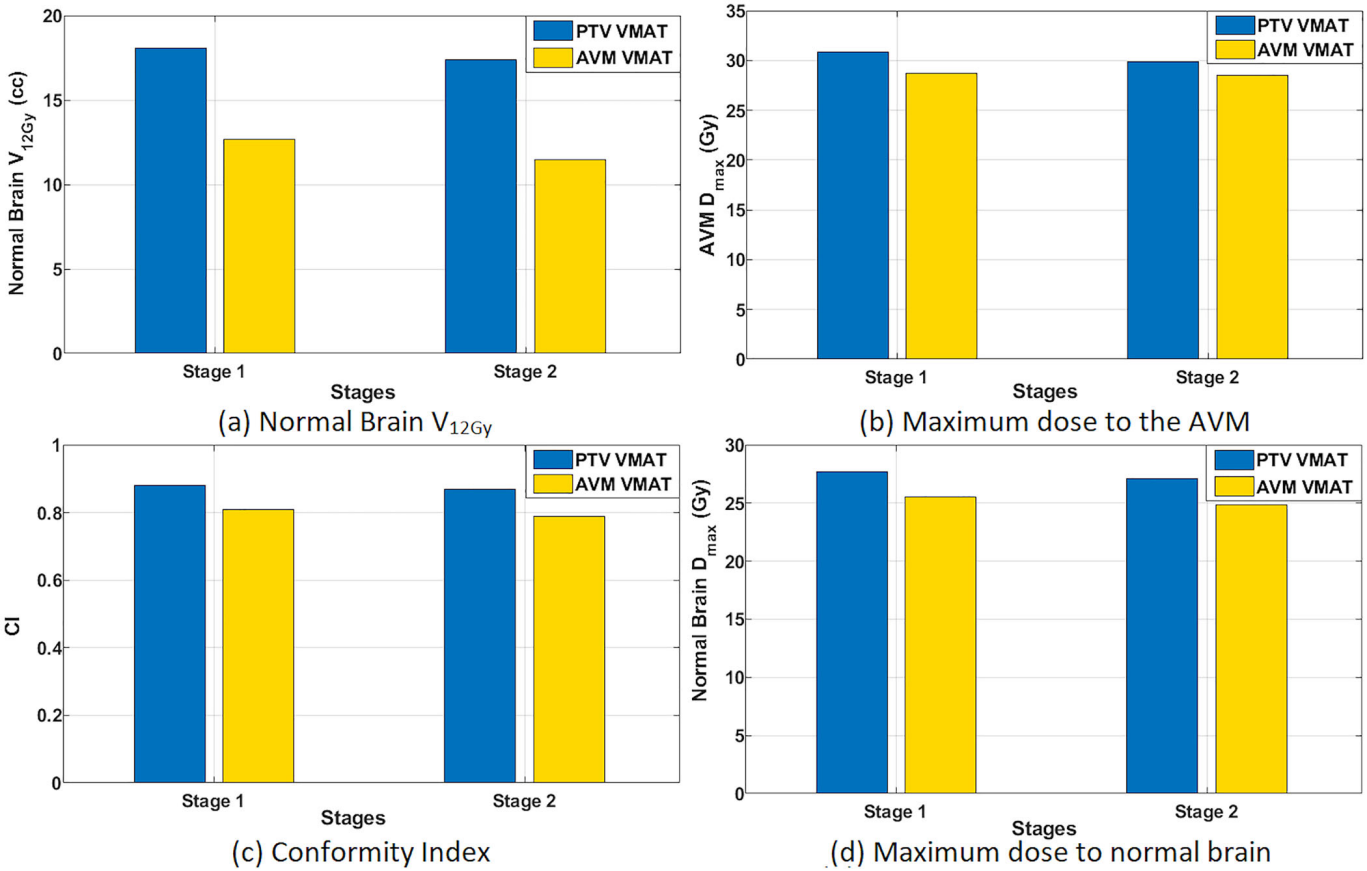
Bar graphs comparing dosimetric parameters for the stages of the AVM‐VMAT and the PTV‐VMAT plans averaged over all patients: (a) normal brain *V*_12Gy_, (b) maximum dose to the AVM, (c) conformity index, and (d) maximum normal brain dose

**FIGURE 8:**
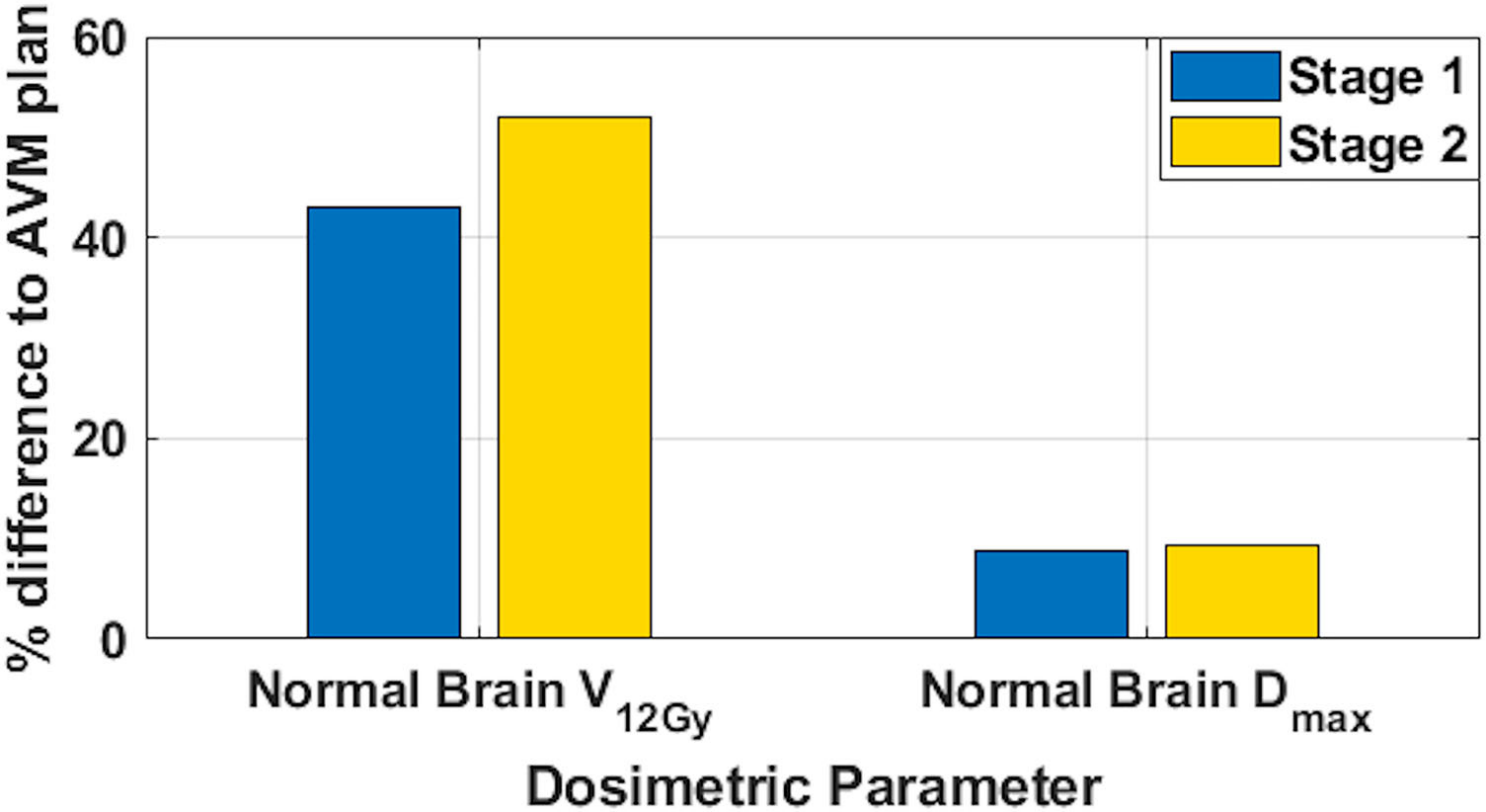
Average percent difference of normal brain *V*_12Gy_ and maximum dose for the PTV‐VMAT plans for all patients with respect to the AVM‐VMAT plans

Figure 7 compares the AVM‐VMAT‐ and PTV‐VMAT‐staged plans for the NB *V*_12Gy_ and maximum dose, AVM maximum dose, and CI averaged per stage. As expected, the NB *V*_12Gy_ is higher for the PTV plans as well as the target's maximum dose and maximum dose to NB; however, conformity is comparable. The differences in NB dose are further highlighted in Figure 8. Overall average NB *V*_12Gy_ increase was 47.5%, and the average increase in NB maximum dose was 9.0%. The conformity to each plan's target was improved by 8.9% and maximum dose inside the AVM was higher for the PTV plans by an overall average of 6.1%.

### 3.2 End‐to‐end testing

#### 3.2.1 Comparison of 1‐setup and 2‐setup film measurements

**FIGURE 9:**
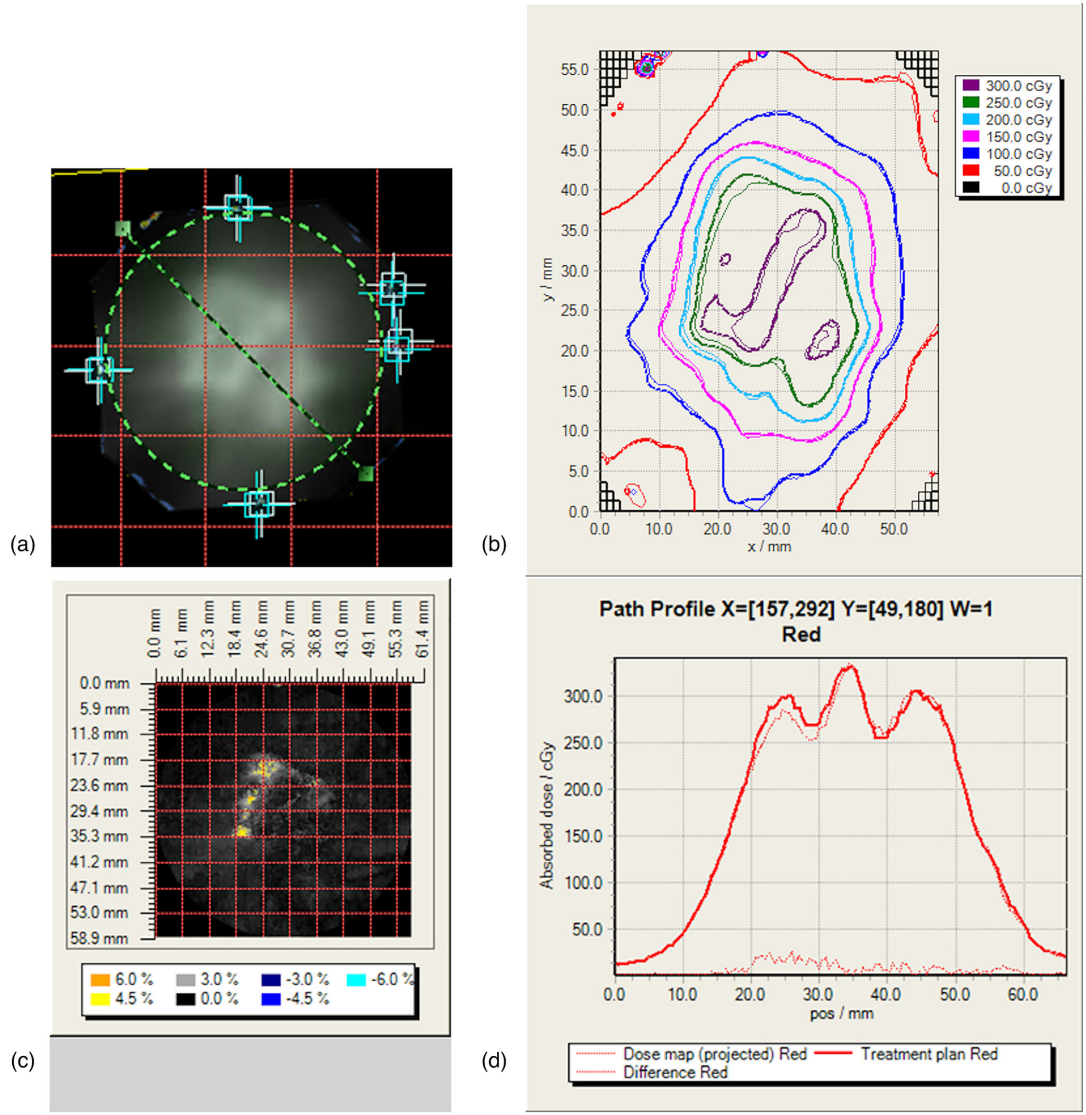
Film‐to film comparison of the 1‐setup and 2‐setup films for patient 3 performed with FilmQA Pro. (a) Alignment of the two films showing the fiducials marked in blue and white, the green dashed line is the line used to extract the profile data and the green dashed circle is the area of the film included in the analysis. (b) Isodose plots for the 1‐setup (thin lines) film and 2‐setup (bold lines) film. (c) Dose difference map in percentage and (d) dose profile plots of the 1‐setup (thin line) and the 2‐setup (bold line). Additionally the difference between the two profiles is also shown.

Figure 9 shows film‐to‐film comparison of the dose distribution of patient 3 measured along the coronal plane using the 1‐setup and 2‐setup method. The alignment of the two films using the fiducial marks is shown in Figure 9a. The dose profiles taken along the line shown in (a), and passing across the junction between the two stages, are plotted in Figure 9d. Isodose plots are compared in Figures 9b, and the dose difference map showing the percentage difference in dose is displayed in Figure 9c. Film‐to‐film comparisons show good agreement in the regions, excluding the junction area, and dose differences of up to 6% can be observed on the fluence maps in the vicinity of the junction.

**FIGURE 10:**
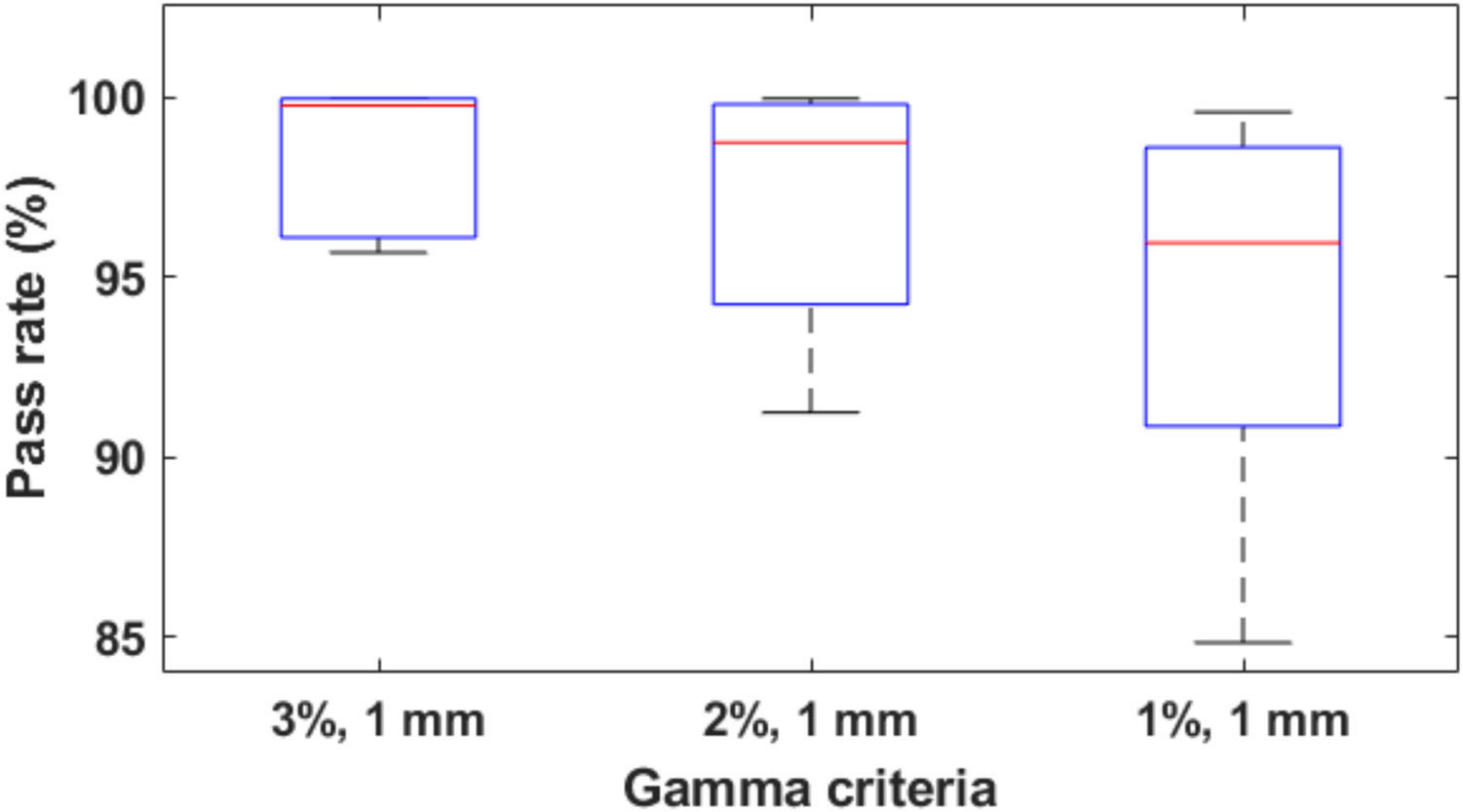
Box plots of the gamma analysis comparing the 1‐setup and 2‐setup film measurements. The box shows the median in red, the 25th and 75th percentile of the data in blue. The vertical lines extend to the highest and lowest values.

Gamma analyses of 3%/1 mm and 2%/1 mm were used to compare the dose distributions from the 1‐setup and 2‐setup measurements. Figure 10 presents a box plot of the median and interquartile of the three gamma criteria used for the analysis of the 10 patients. The average pass rates for the 3%/1 mm, 2%/1 mm, and 1%/1 mm were 98.6%, 97.4%, and 94.9% with a standard deviation of 1.8%, 3.0%, and 4.5%, respectively.

### 3.3 Comparison of film measurements with TPS

**FIGURE 11:**
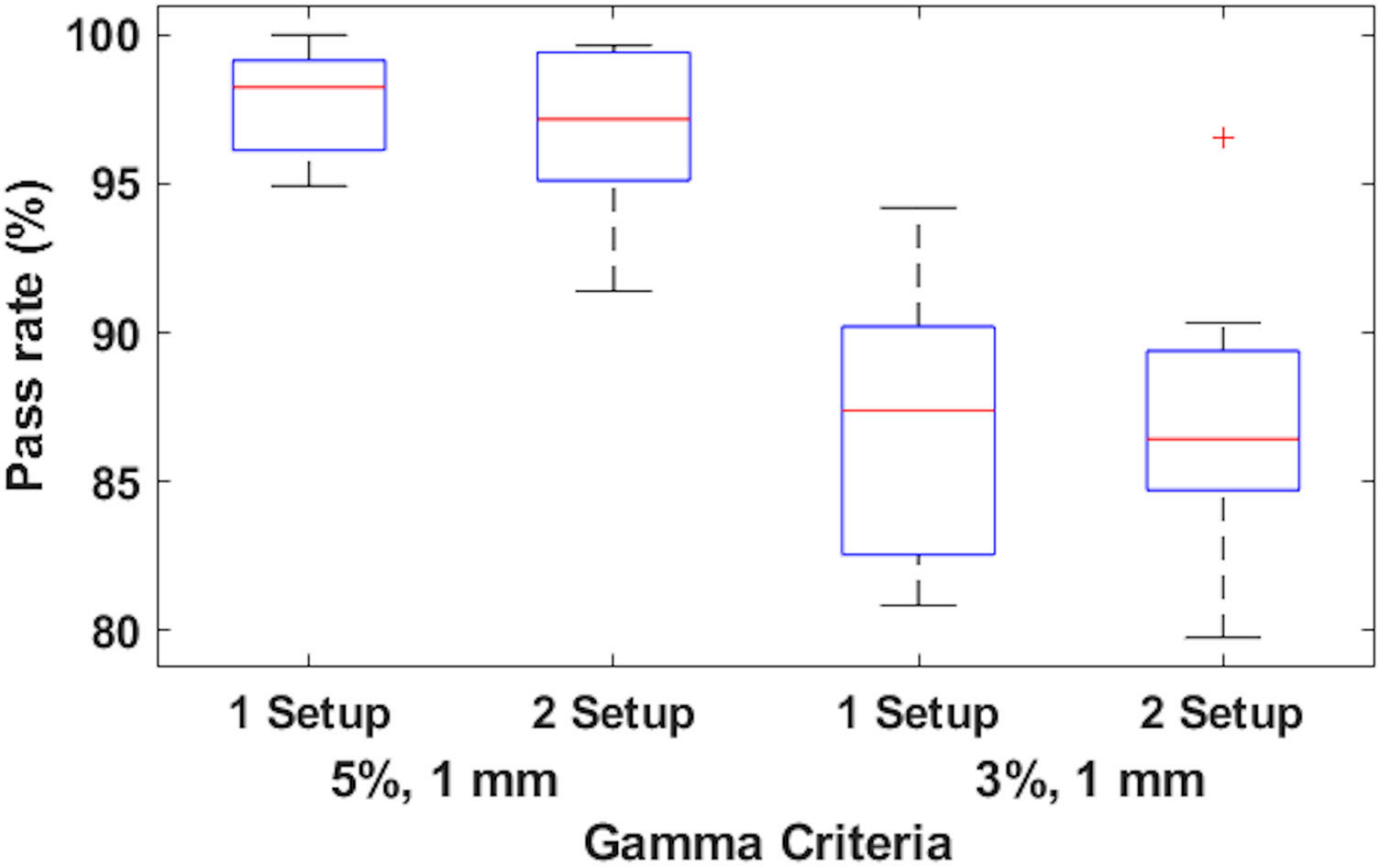
Box plots of the gamma analysis of the 1‐setup and 2‐setup films against the treatment planning system (TPS) dose. The box shows the median in red, the 25th and 75th percentile of the data in blue and the vertical lines extend to the highest and lowest values. Outliers are denoted with a red cross.

A comparison of the 1‐setup and 2‐setup films to the TPS‐calculated dose was also performed. A similar analysis using FilmQA Pro was done for these comparisons, including isodose plot comparisons, dose profiles, and dose difference maps. Dose differences were below 6%, and the largest differences were mainly in the junction region. Figure 11 shows a box plot of the median and interquartile of the two gamma criteria used for both the 1‐setup and 2‐setup films against the TPS dose. The average passing rate (5%/1 mm criterion) for the 1‐setup and 2‐setup film comparisons with TPS calculations was 97.9% and 96.7% with a standard deviation of 1.7% and 2.8%.

### 3.4 Point‐dose measurement

Point‐dose measurements of the PTV‐VMAT volume–staged plans were compared to the point doses calculated by the TPS. The results are summarized in Table 5. The agreement in point dose with the TPS calculation was within 4.0% for all patients with an average of −0.6%. Six of the ten measurements have a discrepancy of more than 2%, which is the tolerance for point‐dose measurement of VMAT plans at our center. The larger uncertainty observed with these measurements is mainly because the measurement point was chosen to be at the junction between the two stages, which exhibits a high‐dose gradient.

**TABLE 5:** Comparison of point‐dose calculation by the treatment planning system (TPS) with measurements using the microDiamond detector

Patient	TPS dose (Gy)	microDiamond dose (Gy)	Difference (%)
1	15.91	15.89	−0.15
2	16.81	17.26	2.66
3	14.81	14.53	−1.92
4	19.12	19.19	0.41
5	19.63	19.43	−1.03
6	18.69	18.12	−3.07
7	18.29	19.02	3.98
8	19.09	18.64	−2.34
9	17.84	17.45	−2.19
10	15.85	15.51	−2.14

## 4 DISCUSSION

In this work, the feasibility of LINAC‐based volume‐staged SRS for the treatment of large AVMs is investigated through a retrospective treatment planning study and dosimetric measurements. Volume‐staged VMAT plans were created and compared with the data available in the literature, and dosimetric errors due to multiple setups were evaluated with end‐to‐end testing. The film‐to‐film comparison done in this work was developed specifically to investigate the dosimetric deviations resulting from two‐stage deliveries involving multiple setups.

The plans that were created using the AVM as the target (AVM‐VMAT) were compared against planning data from GK plans from previous works. These comparisons show that plans with similar dose falloff characteristics to GK's can be achieved with VMAT while keeping the maximum dose under 150% of the prescription dose. GK treatments are commonly prescribed to the 50% isodose line that results in maximum dose inside the target reaching twice the prescription dose.

A comparison of our dosimetric results with published data from three GK centers^13^, ^16^, ^36^ is summarized in Table 6. Franzin et al.^12^ published planning data from volume‐staged treatment plans of 20 AVM patients with median‐staged volumes of 7.8 and 4.7 cm^3^ per stage that were planned with a median prescription dose of 20 Gy. In comparison, the present work used a prescription dose of 20 Gy for all patients with a median‐staged AVM volume of 5.2 and 5.7 cm^3^. The volume of the PTV covered by the prescription isodose from their work (99% and 97%) compares well with ours (97.5% and 97.4%). Their median NB *V*_12Gy_ (24.3 and 15.5 cm^3^) is larger than the median NB *V*_12Gy_ from our plans (10.2 and 10.7 cm^3^).

**TABLE 6:** A comparison of the dosimetric parameters from the AVM‐VMAT plans to Gamma Knife plans obtained from previous publications

Publication	Number of patients	Stages	Prescription (Gy)	Target volume (cm^3^)	Target *D*_max_ (Gy)	Prescription coverage (%)	Normal brain *V*_12Gy_ (cm^3^)	CI
Franzin et al.^12^	20	Stage 1	20 Gy (18–25)	7.8 (2.7–15.8)	40 (36–50)	99 (90–100)	24.3 (14.3–42.7)	NR
		Stage 2	20 Gy (13–24)	4.7 (0.8–10.6)	41 (26–49)	97 (91–100)	15.5 (0.2–32.6)	NR
Nagy et al.^15^	81	Stage 1	17.5 (16–25)	9.7 (3.0–41.1)	NR	NR	NR	0.72 (0.44–0.91)
		Stage 2	17.5 (13–22.5)	9.0 (2.3–24.9)	NR	NR	NR	0.72 (0.4–1)
Gevaert et al.^35^	10	Stage 1	20 Gy	6.2* (0.5–17.2)	38.8* (SD 3.42)	NR	NR	0.8* (SD 0.04)
This work	10	Stage 1	20 Gy	5.2 [7.8] (1.0–19.0)	27.7 [28.7] (25.6–32.9)	97.5 [97.7] (95.5–99.6)	10.2 [12.7] (2.9–34.4)	0.83 [0.81] (0.69–0.91)
		Stage 2	20 Gy	5.7 [7.0] (1.1–17.0)	27.7 [28.5] (24.6–32.6)	97.4 [95.3] (83.5–99.9)	10.7 [11.4] (3.0–21.6)	0.80 [0.79] (0.64–0.91)

*Note*: The values shown per stage are the median, except for those marked by “*” that are mean values, with the range in parenthesis. Mean values for this work are in brackets.

Abbreviations: CI, conformity index; NR, not reported.

Nagy et al.^15^ presented volume‐staged GK plans for 81 patients with a median prescription dose of 17.5 Gy for each stage. The doses were prescribed to the 50% isodose, and the plans had a median CI of 0.72. The median CI of our AVM‐VMAT plans is 0.8, which is slightly higher. In another study, Gevaert et al.^35^ reported planning data from single‐staged treatment plans for 10 AVM cases planned with 20 Gy dose prescription prescribed to the 50% isodose line. The median AVM volume for their plans was 6.21 cm^3^, and the average CI from their work was similar to ours (0.80).

Although GK data was not available for direct comparison with the plans developed in this study, previously published data was used as reference for the dosimetric objectives that should be achieved by the VMAT planning process. Further work in collaboration with a GK center will make direct comparisons of the VMAT and the GK technique possible.

The PTV‐VMAT plans were created on planning targets that were grown from the AVM with a 1 mm margin, following the stereotactic planning protocol at our center. The addition of this margin resulted in a 47.5% increase in the volume of NB that receives 12 Gy or more, when averaged over the 10 patients. This demonstrates that the PTV margin plays a critical role in the volume of NB irradiated with this technique, perhaps more so than in single‐stage SRS plans. Future technological improvements in linear accelerators and imaging may allow for the elimination of the PTV margin for LINAC‐based SRS plans. In a recent publication by Popple et al.,^36^ local tumor control of greater than 90% for multiple brain metastases patients treated with VMAT using a 0 mm PTV margin is reported. Adopting this practice will require a rigorous quality control program to monitor the LINAC's mechanical accuracy^37^ and the accuracy of the patient positioning system.

Although there are no established dose constraints specific to VS‐SRS, the NB *V*_12Gy_ below 10 cm^3^ is recommended for single‐stage SRS treatments to keep the risk of brain radionecrosis to below 10%.^30^ In this work, the *V*_12Gy_ per stage increased with stage volume, which indicates that stage volumes above 10 cm^3^ will likely violate this constraint. One of the strategies adopted by GK centers to lower *V*_12Gy_ per stage increases the number of stages and limits the volume of each stage to 8–10 cm^3^ ^9^, ^10^, ^11^ or lowers the prescription dose.^25^ In this work, no attempt was made to increase the number of stages, and a prescription dose of 20 Gy was used for all the cases.

Imposing additional NB dose constraint on the composite plan may be necessary to ensure the safety of delivering repeated SRS doses to the brain with a short time period in between. A constraint of 0.28 cm^3^ of NB *V*_40Gy_ is recommended by McKay et al.^31^ for cumulative doses of repeated radiotherapy to the brain to limit radionecrosis risk to 10%. It can be seen that some of the PTV‐VMAT plans developed in this work exceeded this constraint, and it is possible that GK plans would also surpass this limit, as the prescription used is generally 20 Gy to the 50% isodose line on the AVM margin.

The plans from this study were verified on an anthropomorphic phantom using film and point‐dose dosimetry. A review of the film data analysis shows that, in general, there was a very good agreement between the 1‐setup and 2‐setup films. This suggests that there is no significant uncertainty introduced by repositioning the phantom to deliver the second stage. The agreement between the TPS dose calculation with either of the films was not as good as the agreements between the 1‐setup and 2‐setup film measurements. This is to be expected as many of the potential systematic error contributions for the film to TPS comparison are eliminated in the film‐to‐film comparison. These factors are (i) the differences in resolution, 1.25 mm for the TPS versus 0.35 mm for the film, (ii) the presence of noise in the film data, (iii) uncertainties due to the scanner's nonuniform response, and (iv) the uncertainties in the selection of the two‐dimensional dose slice by the analysis software.

Point‐dose measurements taken at the junction between the two stages show that the agreement with the TPS calculation was within 4% with an average of −0.6%. Given that the average deviation was less than 1%, it is likely that these discrepancies are mainly due to random error introduced in the high dose gradient region in which these measurements were taken.

## 5 CONCLUSION

This study is the first to plan and evaluate VMAT‐based VS‐SRS treatment plans and to perform an end‐to‐end test to investigate the accuracy of the delivery. Comparison of the planning data from this work with published GK planning data shows that comparable target dose conformality, target dose coverage, and NB *V*_12Gy_ can be achieved with VMAT. Comparison of VMAT and GK VS‐SRS plans calculated on the same planning dataset can further provide more detail on how VMAT compares against the GK technique when applied to VS‐SRS. The finite PTV margin that is routinely applied in LINAC‐based SRS treatments can limit the possible application of LINAC‐based VS‐SRS, and its clinical implementation may depend on future improvements in the delivery and positioning accuracy of LINAC‐based systems.

## AUTHOR CONTRIBUTION

The corresponding author Claudia Mendez is the author of the MSc thesis that this work is based on and performed all measurements and data analysis. Ermias Gete is the MSc supervisor and senior author who contributed the conceptualization, direction and methodology used in this research.

## CONFLICT OF INTEREST

No conflicts of interest.
